# Genetic Characterization of *Canine morbillivirus* (Canine Distemper Virus) Field Strains in Dogs, Chile, 2022–2023

**DOI:** 10.1155/2024/9993255

**Published:** 2024-10-01

**Authors:** Naomi Ariyama, Belén Agüero, Benjamín Bennett, Constanza Urzúa, Felipe Berrios, Claudio Verdugo, Víctor Neira

**Affiliations:** ^1^ Departamento de Medicina Preventiva Animal Facultad de Ciencias Veterinarias y Pecuarias Universidad de Chile, Santiago 8820808, Chile; ^2^ Instituto de Patología Animal Facultad de Ciencias Veterinarias Universidad Austral de Chile, Valdivia 5090010, Chile

## Abstract

Canine distemper virus (CDV) poses a significant threat to dogs and wildlife worldwide, and this study sought to provide an updated genetic characterization of CDV field strains in Chile during 2022–2023. We collected samples from 52 suspected CDV cases in domestic dogs and detected viral RNA through real-time RT-PCR in 28 dogs (54%). Factors like age and vaccination status were determinants of CDV positivity, with young adult and unvaccinated dogs facing a higher infection risk. We isolated CDV from positive samples in VeroDogSLAM cells. From these isolates and direct samples, we obtained sequences and estimated the phylogeny based on gene H. CDV isolates from nasal and conjunctival swabs exhibited cytopathic effects, and sequence analysis unveiled a substantial genetic diversity among the strains. Chilean CDV strains demonstrated a genetic distance to vaccine strains of approximately 10%, antigenic-change-related amino acid substitutions, and novel putative glycosylation sites. In the phylogeny, Chilean CDV field strains clustered into two lineages, Europe/South America-1 and North/South America-4, indicating the emergence of the North/South America-4 lineage in Chile and underscoring the genetic complexity of CDV in the country. Interestingly, certain Chilean viruses shared a close common ancestor with Brazilian and Peruvian viruses, suggesting viral spreading patterns. Further investigations are warranted to comprehend the potential antigenic implications of these genetically diverse CDV strains.

## 1. Introduction

Canine distemper (CD) is a severe infectious-contagious disease caused by *Canine morbillivirus*—canine distemper virus (CDV)—which mainly affects domestic dogs and several wild carnivores [1, 2]. Clinical manifestations of CDV infections in wild and domestic animals include respiratory, digestive, neurologic, and skin disease and a marked immunosuppression associated with lymphoid tissue depletion [1, 3, 4]. A multisystemic disease such as CD may be difficult to diagnose clinically. However, molecular tests on several fluid and tissue samples (e.g., whole blood, urine, and swabs) are useful in early diagnosis [5]. Unfortunately, there is no specific treatment against CD, and the prognosis when the central nervous system is affected is poor [6]. Thus, prevention is essential. To prevent CD, it is necessary a combination of biosecurity measures, such as standard hygiene, isolation or quarantine of diseased dogs, avoiding contact between dogs with unknown health or vaccination status, and immunization measures, such as ensuring colostrum supply and adequate vaccination [7, 8].

CDV is a single-stranded negative-sense RNA virus with a genome of 15.6 kb length that comprises six genes encoding: nucleocapsid (N), phosphoprotein (P), polymerase (L), matrix (M), hemagglutinin (H), and fusion (F) [1]. The hemagglutinin binds the SLAM (signaling lymphocyte activation molecule) and Nectin-4 receptor to infect the host cell [9, 10]. Among all CDV genes, the most variable is the H gene (1824 nt), which is used for phylogenetic analysis [11]. To date, based on this gene, CDV lineages have been described: America-1 (vaccine strains) to -5, Arctic-like, Rockborn-like, Asia-1 to -4, India/Asia-5, Africa-1 and -2, European Wildlife, Europe/South America-1, South America-2, -3, and North/South America-4 [2, 12, 13]. Thus, CDV genetic variability is high, and lineages have different geographical distributions [14, 15].

Recently, the re-emergence of CD has been reported in several countries in vaccinated and nonvaccinated dogs [14, 16–18]. Different factors can contribute to this re-emergence, including increased interaction with wild reservoirs [2–19, 19–21]. Another possible cause of the increased CD occurrence is vaccine failure. Vaccine failure may be associated with improper vaccine administration [7, 22, 23] or due to the escape of divergent CDV strains to the vaccine-induced immune response [21, 24, 25]. Accordingly, a recent study in the USA reported antigenic differences among CDV field strains and between them and the vaccine strains currently used to prevent CD, which may reduce vaccination efficacy [25]. Furthermore, several studies have detected CDV strains divergent from those present in vaccines and amino acid substitutions in the H gene associated with antigenic variation [21, 26–29].

Studies in South America have described highly diverse regional CDV lineages [18, 30–32], evidencing the importance of studying the virus in a local context. In Chile, Salas [33] concluded that at least two lineages, America-1 and Europe/South America-1, are circulating in dogs. However, to date, there are no wide reports on the genetic variability of Chilean CDV strains. This study aims to genetically characterize CDV field strains obtained from naturally infected dogs in Chile.

## 2. Materials and Methods

### 2.1. Sample Collection

To obtain CDV field strains, clinicians from private veterinary clinics from Metropolitana, Valparaiso, and Bio Bio regions took samples from suspected CD cases as routine diagnostic procedures in owned dogs. Dog owners signed an informed consent, and the bioethics committee of the University of Chile, Comité Institucional de Cuidado y Uso de Animales (CICUA), 21438–VET–UCH, approved all sampling procedures. Samples consisted of at least two of the following per dog: conjunctival, nasal, or fecal swabs, whole blood with EDTA, or urine. Sex, breed, age, vaccination status, and clinical signs were documented. All samples were maintained at 4°C for up to 72 h until processed.

From May 2022 to August 2023, we collected 118 samples from 52 CD-suspected animals. Sampling was performed in 15 veterinary clinics and one veterinary diagnostic laboratory (Table S1). Collected sample types included nasal (N, *n* = 4), conjunctival (O, *n* = 10), nasal/conjunctival pooled (ON, *n* = 24), fecal (F, *n* = 40), urine (ORN, *n* = 2), and whole blood (S, *n* = 38).

### 2.2. Viral Detection and Isolation

An aliquot of each sample was used for RNA extraction, and a reverse transcription real-time PCR (RT-qPCR) targeting the N gene of CDV was performed for viral detection. Briefly, for RNA extraction, Trizol reagent (Invitrogen) was used as recommended by the manufacturer, and the purified RNA was stored at −20°C until it was used. Nuclease-free water (Molecular Biology Grade Water, Corning) was used as negative RNA extraction control, and vaccine aliquots (Lederle strain, CANIGEN MHA2PPi/L, Virbac) as positive control. Then, according to a previously described RT-qPCR [34], an 83 bp fragment of a highly conserved region of N gene was amplified using the iTaq Universal Probes One-Step Kit (Bio-Rad, USA) and the following primers and probe: CDV-F: AGCTAGTTTCATCTTAACTATCAAATT, CDV-R: TTAACTCTCCAGAAAACTCATGC, and the PrimeTime probe CDV-Pb: SUN-ACCCAAGAGCCGGATACATAGTTTCAATGC-ZEN/Iowa Black FQ. Cycle threshold (*Ct*) values <40 were considered positive.

Positive conjunctival and nasal samples were inoculated in VeroDogSLAM cells (VDS) as previously described [10] for viral titers increase for sequencing. Dr. Yanagi from Kyushu University, Japan, kindly provided the VDS cells. Approximately 100–200 μl of conjunctival/nasal swabs inoculum was added to VDS in 24-well plates maintained at 37°C and 5% CO_2_ in DMEM (HyClone, Cytiva) (10% FBS, HyClone, Cytiva; G418, 0.4 mg/ml, Gibco) until cytopathic effect (CPE) was observed for up to 7 days post inoculation (dpi). Negative isolation controls consisted of 100 μl of DMEM added to VDS cells instead of the inoculum. After CPE affected >70% of the cells or seven dpi passed without CPE, a further passage was performed in T25 flasks. Viral isolation was confirmed by CPE and RT-qPCR (protocol described for viral detection). The isolation was considered negative when two consecutive passages were CPE negative.

### 2.3. H Gene Sequencing and Phylogenetic Analysis

We conducted the sequencing and phylogenetic analysis of the H gene to identify the CDV genetic lineages circulating in Chile. To amplify and sequence the CDV H gene (1824 bp) from RT-qPCR-positive samples/isolates (*Ct* <26), a conventional RT-PCR (SuperScript III One-Step RT-PCR System with Platinum Taq DNA Polymerase, Invitrogen) was performed as previously described [31]. Then, PCR products were purified using a commercial kit according to the manufacturer's instructions (PureLink Quick Gel Extraction and PCR Purification Combo Kit, Invitrogen), and Sanger sequencing was carried out at the Unidad de Secuenciación y Tecnologías Ómicas, Facultad de Ciencias, Pontificia Universidad Católica de Chile (Santiago, Chile). All the PCR reactions included nuclease-free water (Molecular Biology Grade Water, Cornig) and vaccine strains (Lederle strain, CANIGEN MHA2PPi/L, Virbac) as negative and positive controls, respectively.

Additionally, positive H gene RT-PCR products that failed Sanger sequencing were sequenced using the Native Barcoding Kit 24 V14 (Oxford Nanopore Technologies, UK) in the MinION Mk1C sequencing device (Oxford Nanopore Technologies, UK), according to the manufacturer's instructions. Obtained reads were assembled using Geneious Prime 2024.0.5 (http://www.geneious.com/). First, reads were de novo assembled using the long-read assembly algorithm Flye 2.9.1 [35]. Then, we performed a BLAST search (https://blast.ncbi.nlm.nih.gov/Blast.cgi) of the contigs and assembled the reads using the sequence with the highest identity as reference (CDV008ON mapped to AY386315, CDV040O_P3 to KJ747372, CDV048ORN and CDV051O_P2 to KJ747371) with Minimap2 2.24, option “map-ont” [36].

To determine the genetic diversity and lineages of CDV strains found in our study, a phylogenetic analysis based on the H gene and reference sequences was implemented using Geneious Prime 2024.0.5 (http://www.geneious.com/). All complete H gene sequences (*n* = 845) available on GenBank [37] were retrieved and aligned using MUSCLE [38], including all Chilean sequences available (*n* = 10, accession numbers KU052888–KU052897) and the ones obtained in this study (*n* = 8, accession numbers PP493270–PP493273 and PP850218–PP850221). Duplicate sequences (i.e., sequences with identical residues) were deleted from the alignment, resulting in a final dataset of 804 unique sequences. A maximum likelihood tree was estimated using IQ-TREE v2.1.2 with -m TEST option for nucleotide substitution model selection (selected model according to Bayesian Information Criterion: TVM + F + I + G4) and 1000 ultrafast bootstrap replicates in CIPRES [39, 40]. The tree was visualized using FigTree v1.4.4 (http://tree.bio.ed.ac.uk/software/figtree/). Nucleotide and amino acid *p*-distance matrixes were estimated in Mega X [41]. Also, we identified amino acid mutations of the strains obtained in this study using the Onderstepoort strain as reference (EU143737) and putative glycosylation sites using NetNGlyc-1.0 [42].

### 2.4. Data Analysis

All variables documented during sample collection were analyzed to determine their association with CDV positivity using Pearson's Chi-square test (*α* = 0.15) and univariate logistic regression models (ULRM) to estimate the odds ratio (OR) to CDV positivity. Variables with *p*-values <0.25 in the ULRM were included in a multivariate logistic regression model. Variables with *p*-values >0.1 and that did not affect the significance of other co-variables were removed from the final model. Significant interaction between variables (*α* = 0.1) was also assessed depending on the nature of each one (e.g., age and vaccination status). The goodness of fit of the models was estimated with the Hosmer–Lemeshow test and the McFadden pseudo-R2. Vaccination status was assessed in two ways, using different categorization criteria. To evaluate the overall association between vaccination and CDV positivity, one included dogs of all ages who received at least one vaccine dose or not. The second was only > 6-month-old dogs that have up-to-date vaccines or not, according to 2024 WSAVA guidelines [43]. The latter included only > 6-month-old dogs to evaluate CDV positivity in up-to-date vaccinated dogs. Puppies have maternal-derived antibodies that interfere with vaccine efficacy, requiring multiple doses in a short time to be considered immunized [43]. Since our clinical records only included the last vaccine date and not the complete vaccination calendar, we excluded puppies in the second analysis. Also, we estimated the association between severity of disease and vaccination status using Pearson's *χ*^2^ test (*α* = 0.05). All statistical analyses were performed using the R software (version 4.1.3) [44] in RStudio (version 2023.09.0) [45].

## 3. Results

Details of all clinical records, samples, and RT-qPCR results of the cases included in this study are available in Table S1 and Table S2. Regarding the clinical records, most dogs were mixed breed, and the sex ratio was 1.3:1 (female:male). The age of the dogs varied from 1 month to 5 years and 11 months, with an average age of 1 year and 4 months and a median age of 6 months. The predominant clinical signs were ocular and digestive symptoms. Nine dogs had severe disease requiring hospitalization (Table S1).

From 52 CD-suspected dogs, 52 out of 118 (44%) samples resulted in positive for CDV genetic material. The sample types with the higher sample positivity rates were the pooled or individual conjunctival and nasal swabs, with 20 out of 38 (53%) positive samples (Table S2 and Figure S1). *Ct* values ranged from 16 to 39. Fecal and blood samples showed the lowest *Ct* values, 16 and 17, respectively (Table S2). Positive cases corresponded to those with at least one positive sample to the N gene RT-qPCR. Thus, 28 dogs out of 52 were CDV-positive cases, resulting in a positivity rate of 54%.

**Figure 1:**
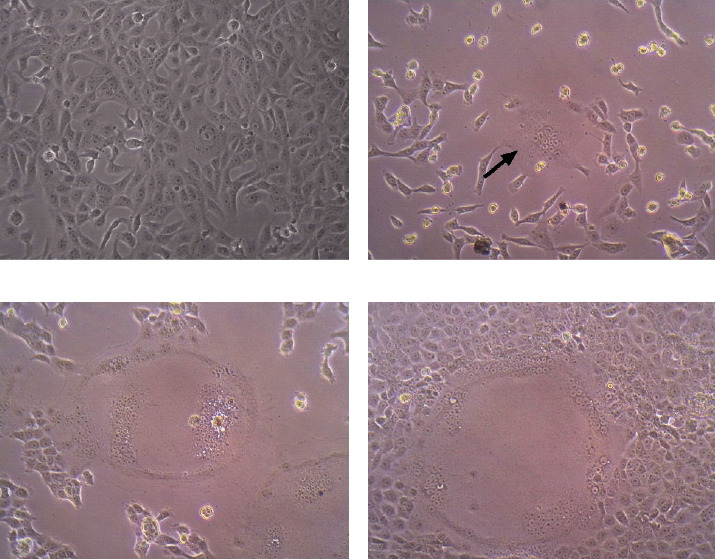
CDV isolation in VeroDogSLAM cells (VDS). (a) Control VDS without inoculation. (b) VDS inoculated with a conjunctival and nasal swabs pooled sample (CDV045ON) 24-h postinoculation; the arrow label indicates a syncytium with centric nuclei. (c) VDS inoculated with CDV045ON 2 days post inoculation (dpi); in the center, a syncytium with peripheric nuclei is shown. (d) VDS inoculated with CDV045ON 3 days postinoculation; a larger syncytium with peripheric nuclei is shown.

Regarding isolation, 13 out of 18 (72%) inoculated samples (nasal and/or conjunctival) in VDS cells were positive for RT-qPCR. We observed CPE in seven isolates (39% isolation rate) with syncytia formation as early as 24 h postinoculation (Table 1, Figure 1a–d).

Statistical analysis results are summarized in Tables 2 and 3; 47 out of the 52 dogs' clinical records were available (Table S1). According to univariate analysis, factors statistically associated with CDV positivity were age, breed, location, vaccination status, ocular, respiratory, digestive, and neurological signs (Table 2). Regarding vaccination, 23 out of 43 owners declared that their dogs had received at least one vaccine dose in their lives. Among these 23 vaccinated dogs, 11 (48%) were CDV-positive. In the case of unvaccinated dogs, 15 out of 20 (75%) were CDV-positive. In this sense, univariate models showed that the odds of a dog being CDV positive were three times higher when not vaccinated (95% CI 1.16–9.23, *p*  < 0.1). In contrast, excluding puppies, unvaccinated/outdated CDV-positive dogs were 9 out of 15 (60%), and 5 out of 9 (56%) were up-to-date vaccinated. Vaccination and CDV positivity in >6-month-old dogs were not statistically associated (*p*-value = 1) (Figure S2).

Another relevant factor in univariate models was age. The positivity according to age stage was the following: puppies: 13 out of 22 (59%), young adults: 8 out of 9 (89%), and mature adults: 6 out of 16 (38%). The odds of a dog being CDV positive were 13 times higher in young adult dogs (95% CI, 1.81–281.06.16, *p*  < 0.05). In contrast, the final regression model showed a significant association only between clinical signs and CDV positivity (Table 3). Finally, when we evaluated the association between severe disease presentation and vaccination status, these were independent (*χ*^2^ = 0.2983, df = 1, *p*-value = 0.59), indicating that vaccination may not be a protective factor against severe disease.

**Figure 2:**
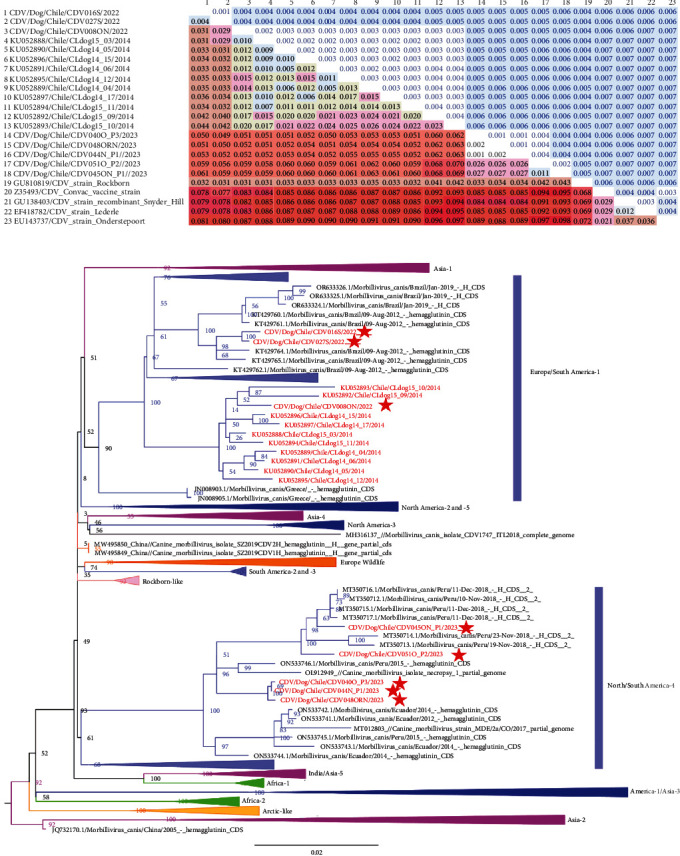
Genetic analyses of Chilean CDV field strains. (a) Genetic divergence of Chilean and vaccine CDV strains (*p*-distance matrix). The number of base differences per site between sequences is shown; standard error estimates are shown in blue above the diagonal. (b) IQ-Tree ML phylogenetic tree; bootstrap values are labeled in the nodes. CDV lineages are labeled; Chilean strains are shown in red; the red stars specify sequences obtained in this study.

We sequenced eight strains, four from original samples and two from isolates (Table 1). The obtained sequences showed a pairwise identity of 96% at nucleotide and amino acid levels. Compared to the most used America-1 vaccine strains (Z35493 Convac, GU138403 Snyder_Hill, EF418782 Lederle, and EU143737 Onderstepoort), the mean distance to all Chilean strains was near 9% (8.73%) and 10% (9.55%) in nucleotides and amino acidic sequences (Figure 2a).

According to the phylogeny, Chilean CDV viruses belong to the Europe/South America-1 and North/South America-4 lineages (Figure 2b). Three viruses, CDV008ON (PP850218), CDV016S (PP493270), and CDV027S (PP493271), grouped in two clades within the Europe/South America-1 lineage and shared a common ancestor with Brazilian viruses from 2012 (KT429764–KT429765) and Chilean viruses from 2014 (KU052892–KU052893). In the North/South America-4 lineage, the Chilean viruses CDV040O_P3 (PP850219), CDV044N_P1 (PP493272), CDV045ON_P1 (PP493273), CDV048ORN (PP850220), and CDV051O_P2 (PP850221) were closely related to viruses collected in Peru during 2018 (MT350712–MT350717) and 2015 (ON533746), suggesting a common origin of Peruvian and the Chilean viruses. Also, a virus obtained in 2021 from a fox in the United States (OL912949) grouped within this Peruvian–Chilean cluster. However, the CDV040O_P3 (PP850219), CDV044N_P1 (PP493272), and CDV048ORN (PP850220) formed a paraphyletic branch. The viruses found in this study were genetically distant from the Vaccine/North America-1/Asia-3 containing the classical vaccine strains. On the contrary, the Rockborn-like strains, including vaccine strains from Asian countries, are genetically closer to the Chilean CDV field strains than the standard America-1 vaccine strains.

Mutations identified in Chilean field strains obtained in this study are summarized in Table S3. We found some amino acid mutations in residues with known function/effect: D238Y (all excepting CDV016S and CDV027S) and R241G (excepting CDV008ON) related to antigenic change in a neutralizing epitope; A367V (all sequenced strains), G376N (all), and T386S (all from the North/South America-4 lineage) are putative antigenic residues; Nectin-4 binding residues: S460L (all), I510L (all), and I522V (all North/South America-4 lineage); SLAM-binding residue: M500R (all); lineage related mutations and SLAM-binding residue: S530N (CDV040O_P3, CDV044N_P1, and CDV048ORN), S530D (CDV045ON_P1 and CDV051O_P2); H549Y (all) is a host-related mutation and a SLAM-binding residue.

We identified N-linked glycosylation sites frequently described in worldwide CDV lineages 19–21, 149–151, 309–311, 391–393, 422–424, 456–458, 587–589, 603–605, excepting 584–586, (12) in all the sequences obtained in this study (Figure S3). The 309–311 site was absent in CDV016S and CDV027S. Unusual putative glycosylation sites were 261–263 (CDV008ON), 301–303 (CDV045ON_P1), and 542–544 (CDV016S and CDV027S).

## 4. Discussion

We found highly divergent Chilean CDV field strains from vaccinated and unvaccinated dogs from the Metropolitana and Valparaiso regions. Almost 40% of the identified positive cases have received at least one vaccine dose against CDV. Among the available CDV vaccines in Chile (Table S4), the most used are Nobivac, Canigen, Vanguard, and Recombitek. The first two contain America-1 lineage-derived strains (Onderstepoort and Lederle), and the remaining do not declare from which lineage they are (N-CDV and CDP258 strains). CDV vaccine-induced protective antibodies can last up to 5 years postvaccination [23]. However, those antibodies are measured using viral neutralization tests against reference viral strains that may differ from circulating field strains. Furthermore, low cross-neutralization between America-1 vaccine strains and wild-type strains has been reported in the United States [25]. These data are worrisome since the increase in CD cases among vaccinated animals was reported in several countries [14, 16–18, 46–48].

Potential variables associated with CDV positivity were analyzed (Tables 2 and 3) regarding the OR estimated with the final multivariate model; although not statistically, a clinically significant variable such as age showed that young adults (OR: 6.9) (6 months–2 years old) had a higher OR than puppies (OR: 2.9) (Table 3). Also, half of the positive cases were older than 6 months of age. Another clinically significant variable, vaccination, resulted in a protective factor against CDV positivity (OR: 0.3). Location and breed were not considered clinically significant due to the Metropolitana region and mixed breed overrepresentation, respectively. It may be necessary to include other factors (e.g., number of vaccine doses, nutritional status, colostrum intake, and indoor status) not recorded in our study to improve the final model.

Maternally derived antibodies interfere with vaccine efficacy in puppies <4–6 months of age. Thus, vaccination in puppies <6 months is repeated every 2–3 weeks (from 1.5 to 4 months of age). Accordingly, WSAVA guidelines recommend one CDV vaccine dose in dogs >6 months and triennial revaccination with core vaccines [43]. Therefore, we performed an additional analysis excluding puppies that resulted in vaccination and CDV positivity not statistically associated (*p*-value = 1) (Figure S2). Also, cases with severe disease resulted independent of vaccination status (*p*  > 0.5), increasing concerns about vaccine efficacy against Chilean field strains. However, although CDV positivity in >6-month-old dogs and disease severity are not statistically associated with vaccination status, our data reflects some grade of vaccine protection on the variables' effect sizes and the lower positivity risk in univariate logistic regression (Tables 2 and 3). Furthermore, the sample size may influence the significance of the tests [49], which may improve with a larger group of cases. A limitation of our analysis is the lack of maternal and neutralizing antibodies assessment to ensure proper vaccination protocols during routine clinical practice for the cases included in this study; the vaccination effect may be underestimated. Thus, given its clinical relevance, vaccination is highly recommended to prevent or reduce the severity of CD and other infectious diseases typically included in multivalent canine vaccines.

To our knowledge, this is the first report of Chilean CDV field strain isolation (Table 1). Seven isolates from pooled or individual conjunctival and nasal swabs showed CPE (39% isolation rate). Some as early as 24-h postinoculation, similar to previous reports on CDV field and vaccine strains isolation in VDS cells [47, 50]. However, previous studies showed lower isolation rates than ours, 1 out of 18 (6%) positive nasal swabs [51], and 1 out of 7 (14%) positive dog tissues [52]. These Chilean CDV isolates could be useful for further antigenicity studies.

From the sequences obtained in this study, five out of eight corresponded to vaccinated dogs showing high diversity, with mean distances of 4% among them at the nucleotide and amino acid levels. The isolate CDV045ON_P1 and CDV051O_P2 showed the highest genetic distance among all Chilean viruses (up to 7%) (Figure 2a). Compared to vaccine strains, except for the Rockborn, the genetic distance of Chilean strains was near 10%. Accordingly, Rockborn strains have shown high cross-neutralization levels against multiple CDV strains, including field strains of the United States [25]. However, Rockborn vaccines were withdrawn from the market in the 1990s due to security concerns on residual virulence and are not recommended for use [53].

Regardless of the low number of CDV sequences available from Chile (*n* = 18), we found a novel lineage for Chile, the North/South America-4, and other viruses from the Europe/South America-1 lineage. The North/South America-4 cluster included the CDV040O_P3 (PP850219), CDV044N_P1 (PP493272), CDV045ON_P1 (PP493273), CDV048ORN (PP850220), CDV051O_P2 (PP850221), and viruses from the USA, Colombia, Ecuador, and Peru. Interestingly, a virus from a wild host from the USA (OL912949) grouped within this Peruvian–Chilean subcluster. Also, this lineage potentially infects wild species and has a wide distribution across the Americas [13, 54]. Despite the lack of CDV sequences from Chilean wild hosts, CDV outbreaks and antibodies presumably derived from the spillover transmission from domestic dogs have been reported on these [55–58]. Thus, the potential risk and impacts of CDV interspecies transmission in the domestic wildlife interfaces and spillover from domestic dogs to wildlife needs further research in Chile.

The CDV008ON (PP850218), CDV016S (PP493270), and CDV027S (PP493271) strains, grouped within the Europe/South America-1, share a common ancestor with Brazilian viruses from 2012 (KT429764- KT429765) and other Chilean sequences (up to 4.4% of nucleotide distance) collected in 2014 (KU052892-KU052893) in Los Rios region (39°S), more than 700 km from the Metropolitana and Valparaiso regions (33°S). Thus, despite belonging to the same lineage, these Chilean viruses differ genetically. Also, considering the particular geographical length of the Chilean territory, the genetic diversity of CDV circulating in dogs may be higher, and further studies on this situation are necessary. The lack or scarcity of CDV sequences from Chilean wild and domestic hosts and other South American countries, especially those sharing boundaries with Chile (i.e., Bolivia), are limitations of the phylogenetic analyses.

Mutation is one of the main evolutionary drivers of RNA viruses [59]. We detected several amino acid substitutions related to host species and antigenic variation in the H gene sequences obtained (Table S3). Regarding antigenicity, we identified the D238Y and R241G substitutions in all the Chilean North/South America-4 lineage viruses. These substitutions elicit antigenic change in a neutralizing epitope of CDV H protein [26]. Similar to a recent report in Brazil, we identified mutations in putative antigenic residues A367V, G376N, and T386S (the last only in North/South America-4 lineage) that could suggest antigenic variation [28]. These substitutions suggest that Chilean CDV strains may be antigenically divergent, especially the North/South America-4 lineage. Thus, we may expect differences in vaccine-induced immune response against local strains. In addition, we found unusual putative N-linked glycosylation sites on Chilean strains (Figure S3). Glycosylation in viral surface proteins may enhance immune evasion or viral entry and needs further investigation [60, 61].

There is controversy about residues 530R/N/D and 549H as indicators of interspecies transmission [62]. According to Nikolin et al. [63], these two substitutions are lineage-related rather than host-related. On the other hand, Bhatt et al. [12] observed similar patterns in 530 as Nikolin et al. [63]; however, they did find a trend of 549H variants in non-canid hosts [12]. Accordingly, the Chilean sequences obtained, which were all from canid hosts, showed the H549Y substitution. Also, 530 and 549 are SLAM-binding residues that may impact viral fusion activity [64]. Finally, substitutions found in S460L, I510L, and I522V (the last only in North/South America-4 lineage) may affect viral binding activity in the cell receptor Nectin-4, especially S460L that implies a polarity change in this residue. SLAM and Nectin-4 sequential binding are essential for viral transmission, and epithelial infection is for clinical signs and disease [65, 66]. Thus, changes in the binding site of the epithelial receptor Nectin-4 may alter disease progression.

A limitation of our study is that most of the samples, including the positive ones, were collected in the Metropolitana region. Nevertheless, the Metropolitana and Valparaiso regions are the most human and dog-populated in the country [67, 68]. The Chilean dog population is approximately 7 million and has a median density of 12 dogs/km^2^ [68]. Valparaiso and Metropolitana regions have up to 2000 and 7000 dogs/km^2^ in some districts, respectively [68]. Consequently, sampling directed to these regions may represent well the national situation. Another limitation of our analysis is the lack of whole genome sequences. As previously reported, recombination causes a discrepancy in tree topologies among different CDV genome segments and between the H gene and concatenated whole genome phylogenies, leading to lineage misclassification [69–71]. However, the H gene encodes the major antigenic protein of CDV and remains a good indicator of genetic and antigenic variability [24, 72, 73].

## 5. Conclusions

In conclusion, clinicians should perform a CDV-antibodies assessment during routine vaccination programs to ensure appropriate immune protection. Our results suggest that the genetic divergence of Chilean field strains may imply antigenic changes. Therefore, CDV vaccine-induced antigenicity and immunity against Chilean field strains need to be assessed.

## Figures and Tables

**Table 1:** Summary of CDV isolation and H gene sequencing, Chile, 2022–2023.

ID	Sample type	*Ct*	H gene PCR	Sanger seq	Nanopore seq	Isolation	CPE HPI	*Ct* P1	*Ct* P2	*Ct* P3
CDV001	O	21.2	Yes	Partial	Failed	No	—	33.2	—	—
CDV002	ON	22.2	Yes	Failed	Failed	No	—	30.6	33.2	39.1
CDV005	ON	23	Yes	Failed	Failed	No	—	30.9	30.3	33.4
CDV008	ON	25.3	Yes	Failed	Yes	No	—	29.1	31.9	33.2
CDV012	ON	26.8	—	—	—	No	—	34.4	36.1	—
CDV015	N	22	Yes	Failed	Failed	Yes	48	20.1	—	—
CDV016	ON	21.3	No	—	—	Yes	48	22	16.4	—
CDV016	S	18	Yes	Yes	—	—	—	—	—	—
CDV018	ON	30.1	—	—	—	No	—	>40	>40	>40
CDV019	ON	26.3	Yes	Failed	Failed	Yes	48	22.9	22.1	17.9
CDV023	ON	36.9	—	—	—	No	—	>40	38.6	>40
CDV027	S	19.3	Yes	Yes	—	—	—	—	—	—
CDV038	N	30.2	—	—	—	No	—	>40	—	—
CDV038	O	28.6	—	—	—	No	—	28.6	31.4	—
CDV040	O	25.2	Yes	Failed	Yes	Yes	48	30.9	32	24.8
CDV041	O	28.4	—	—	—	No	—	30.9	35.9	38.5
CDV044	N	25.5	Yes	Yes	—	Yes	48	25.5	27.2	—
CDV045	ON	37	Yes	Yes	—	Yes	24	29.3	32.1	23.4
CDV048	ORN	24.8	Yes	Failed	Yes	—	—	—	—	—
CDV049	ON	35.6	—	—	—	No	—	>40	—	—
CDV051	O	23.2	Yes	Failed	Yes	Yes	24	20.4	—	—

*Note:* CPE HPI, hours postinoculation when cytopathic effect was evidenced; *Ct* P1-3, cycle threshold on each passage; N, nasal swab; O, conjunctival swab; ON, pooled nasal and conjunctival swabs; S, blood with EDTA.

Abbreviation: *Ct*, cycle threshold.

**Table 2:** Summary of variables potentially associated with CDV positivity (*n* = 47).

Variable	Total (%)	Positive	Negative	*χ* ^2^ (*α*: 0.1)	Effect size	OR (*α*: 0.25)		95% CI
Sex	—	—	—	*χ* ^2^ = 0.00 *p* = 1.00	0.02	—	—	—
Female	24 (51)	14	10	—	—	1.63	*p* = 0.28	0.69–4.11
Male	23 (49)	13	10	—	—	0.89	*p* = 0.85	0.26–3.04
Age	—	—	—	*χ* ^2^ = 6.27 *p* = 0.04 ^*∗*^	0.37 ^*∗*^	—	—	—
Puppy (<6 months)	22 (47)	13	9	—	—	2.41	*p* = 0.19 ^*∗*^	0.66–9.47
Young adult (6 m–2 years)	9 (19)	8	1	—	—	13.33	*p* = 0.03 ^*∗*^	1.81–281.06
Adult (>2 years)	16 (34)	6	10	—	—	0.60	*p* = 0.32	0.20–1.62
Breed	—	—	—	*χ* ^2^ = 0.41 *p* = 0.52	0.14	—	—	—
Known breed	13 (28)	6	7	—	—	0.53	*p* = 0.34	0.14–1.93
Mixed	34 (72)	21	13	—	—	1.62	*p* = 0.17 ^*∗*^	0.82–3.31
Location	—	—	—	*χ* ^2^ = 1.68 *p* = 0.19	0.24	—	—	—
Metropolitana	34 (72)	22	12	—	—	1.83	*p* = 0.08 ^*∗*^	0.92–3.83
Valparaiso	13 (28)	5	8	—	—	0.34	*p* = 0.24 ^*∗*^	0.09–1.25
Vaccination status	—	—	—	*χ* ^2^ = 2.27 *p* = 0.13	0.28 ^*∗*^	—	—	—
No	20 (47)	15	5	—	—	3.00	*p* = 0.07 ^*∗*^	1.16–9.23
Yes (at least one dose)	23 (53)	11	12	—	—	0.31	*p* = 0.03 ^*∗*^	0.08–1.08
Ocular signs	—	—	—	*χ* ^2^ = 6.79 *p* = 0.01 ^*∗*^	0.44 ^*∗*^	—	—	—
No	16 (36)	5	11	—	—	0.45	*p* = 0.14 ^*∗*^	0.14–1.25
Yes	29 (64)	22	7	—	—	6.91	*p* = 0.01 ^*∗*^	1.88–29.20
Respiratory signs	—	—	—	*χ* ^2^ = 4.59 *p* = 0.03 ^*∗*^	0.37 ^*∗*^	—	—	—
No	20 (44)	8	12	—	—	0.67	*p* = 0.38	0.23–1.62
Yes	25 (56)	19	6	—	—	4.75	*p* = 0.02 ^*∗*^	1.37–18.24
Digestive signs	—	—	—	*χ* ^2^ = 1.21 *p* = 0.27	0.22	—	—	—
No	10 (22)	4	6	—	—	0.67	*p* = 0.53	0.17–2.33
Yes	35 (78)	23	12	—	—	2.88	*p* = 0.15 ^*∗*^	0.69–13.20
Neurological signs	—	—	—	*χ* ^2^ = 4.04 *p* = 0.05 ^*∗*^	0.35 ^*∗*^	—	—	—
No	23 (51)	10	13	—	—	0.77	*p* = 0.53	0.33–1.75
Yes	22 (49)	17	5	—	—	4.42	*p* = 0.02 ^*∗*^	1.15–17.41
Severe disease	—	—	—	*χ* ^2^ = 0.00 *p* = 1.00	0.00	—	—	—
No	30 (67)	18	12	—	—	1.50	*p* = 0.28	0.73–43.19
Yes	15 (33)	9	6	—	—	1.00	*p* = 1.00	0.28–3.67

*Note:* Association and risk were evaluated through Pearson's *χ*^2^ test (*α*: 0.1), Cohen's *ω* for the effect size (0.1 small,  ^*∗*^ 0.3 medium, 0.5 large) and univariate logistic regression analysis (OR: >1 higher risk, <1 protective factor).

**Table 3:** Multivariate logistic regression model of variables associated with CDV positivity.

I. Initial multivariate logistic regression model of variables associated with CDV positivity. *α*: ^*∗*^ 0.1; McFadden pseudo *R*^2^ = 0.6052 *p*-value = 0.0002; Hosmer–Lemeshow *χ*^2^ = 1.6355 *p*-value = 0.9902; AIC = 44.039							
**Variable**	**Coefficient**	**SE**	***z***	***p*-Value**			

Vaccination	−2.5835	1.5851	−1.63	0.1031			
Breed	0.3526	2.103	0.168	0.8669			
Location	−5.0601	2.6035	−1.944	0.0519 ^*∗*^			
<6 months	3.8778	2.123	1.827	0.0678 ^*∗*^			
≤2 years	10.7296	7.3213	1.466	0.1428			
Ocular signs	3.1593	1.5219	2.076	0.0379 ^*∗*^			
Neurological signs	1.4693	1.5947	0.921	0.3568			
Respiratory signs	5.7482	3.1282	1.838	0.0661 ^*∗*^			
Digestive signs	8.7155	5.7877	1.506	0.1321			
Severity	−4.2311	2.4271	−1.743	0.0813 ^*∗*^			
Constant	−9.4765	5.6577	−1.675	0.0939 ^*∗*^			

**II. Final multivariate logistic regression model of variables associated with CDV positivity. *α*:** ^*∗*^**0.1; McFadden pseudo *R*^2^ = 0.4507 *p*-value = 0.0007; Hosmer–Lemeshow *χ*^2^ = 11.812 *p*-value = 0.1598; AIC = 46.66**							

**Variable**	**Coefficient**	**SE**	***z***	***p*-Value**	**OR**	**95% CI**	

Vaccination	−1.15	1.151	−0.999	0.3176	0.3165	0.0001	0.3400
<6 months	1.083	1.373	0.789	0.4303	2.9524	0.0254	2.7979
≤2 years	1.93	1.813	1.065	0.2869	6.8927	0.2302	66.5395
Ocular signs	2.092	1.054	1.986	0.0471 ^*∗*^	8.1050	0.3002	560.4896
Neurological signs	1.812	1.26	1.438	0.1503	6.1242	1.2002	88.4884
Respiratory signs	1.75	1.016	1.722	0.0851 ^*∗*^	5.7553	0.8567	55.9788
Digestive signs	2.432	1.372	1.773	0.0763 ^*∗*^	11.3815	1.0087	292.6173
Constant	−4.488	1.989	−2.256	0.0241 ^*∗*^	0.0112	0.6479	120.5520

*Note:* I. Initial multivariate model including all variables with univariate regression *p*-values <0.25.

II. Final multivariate model including variables with *p*-values <0.1 from the initial model.

## Data Availability

The data that support the findings of this study are available from the corresponding author upon reasonable request.

## Supporting Information

Supporting Information Supporting informations Table S1, S2, S3, S4, and Figures S1, S2, and S3 (see legends below) are included for detailed information of the reported canine distemper (CD) cases (Table S1), amino acid substitutions on the sequences (Table S3), CDV vaccines are available in Chile (Table S4), canine distemper virus (CDV) RT-qPCR positivity (Figure S1), CDV positivity in >6-month-old dogs (Figure S2), putative N-linked glycosylation sites in the H gene of Chilean CDV strains (Figure S3). Table S1. Summary of clinical records of suspected CD cases included in this study, Chile, 2022−2023. Table S2. Vaccination status and RT-qPCR results of the suspected CD cases included in this study, Chile, 2022−2023. Table S3. Amino acid substitutions on Chilean CDV field strains 2022−2023 compared to Onderstepoort reference strain, number of strains with the mutation, function of the specific site, and parsimony informative sites. Table S4. Vaccines against CDV are available in Chile according to the Agricultural and Livestock Service (SAG), May 2024. Figure S1. CDV RT-qPCR positivity according to sample type in suspected CD cases, Chile, 2022−2023. Overall CDV positivity of 118 samples from 52 suspected CD cases included in this study; the conjunctival/nasal category included pooled and individual samples. Figure S2. CDV positivity according to vaccination status in >6-month-old dogs, Chile, 2022−2023. According to Fisher's exact test (*p*-value = 1), CDV positivity is not associated with vaccination status in >6-month-old dogs. Figure S3. Putative N-linked glycosylation sites predicted in Chilean and Onderstepoort CDV strains amino acid sequences using NetNGlyc 1.0. Asn-Xaa-Ser/Thr motifs are highlighted in blue. Asparagines predicted to be N-glycosylated are highlighted in red. Glycosylation sites are frequently found in different CDV lineages, according to Bhatt et al. [12], are labeled in black rectangles.
