# Supplementary material for: Genetic Characterization of Canine morbillivirus (Canine Distemper Virus) Field Strains in Dogs, Chile, 2022–2023
Source: Transbound Emerg Dis. 2024 Oct 1;2024:9993255. doi: 10.1155/2024/9993255 (PMC12017005; doi:10.1155/2024/9993255)
Supplement: Supporting Information — s Table S1, S2, S3, S4, and Figures S1, S2, and S3 (see legends below) are included for detailed information of the reported canine distemper (CD) cases (Table S1), amino acid substitutions on the sequences (Table S3), CDV vaccines are available in Chile (Table S4), canine distemper virus (CDV) RT-qPCR positivity (Figure S1), CDV positivity in >6-month-old dogs (Figure S2), putative N-linked glycosylation sites in the H gene of Chilean CDV strains (Figure S3). Table S1. Summary of clinical records of suspected CD cases included in this study, Chile, 2022−2023. Table S2. Vaccination status and RT-qPCR results of the suspected CD cases included in this study, Chile, 2022−2023. Table S3. Amino acid substitutions on Chilean CDV field strains 2022−2023 compared to Onderstepoort reference strain, number of strains with the mutation, function of the specific site, and parsimony informative sites. Table S4. Vaccines against CDV are available in Chile according to the Agricultural and Livestock Service (SAG), May 2024. Figure S1. CDV RT-qPCR positivity according to sample type in suspected CD cases, Chile, 2022−2023. Overall CDV positivity of 118 samples from 52 suspected CD cases included in this study; the conjunctival/nasal category included pooled and individual samples. Figure S2. CDV positivity according to vaccination status in >6-month-old dogs, Chile, 2022−2023. According to Fisher's exact test (p-value = 1), CDV positivity is not associated with vaccination status in >6-month-old dogs. Figure S3. Putative N-linked glycosylation sites predicted in Chilean and Onderstepoort CDV strains amino acid sequences using NetNGlyc 1.0. Asn-Xaa-Ser/Thr motifs are highlighted in blue. Asparagines predicted to be N-glycosylated are highlighted in red. Glycosylation sites are frequently found in different CDV lineages, according to Bhatt et al. [12], are labeled in black rectangles. [file 9993255.f1.docx]

**Supplementary Material**

**Table S1.** Summary of clinical records of suspected CD cases included in this study, Chile, 2022-2023.

|  |  |  |  |  |  |  |  | Clinical signs | | | | |
| --- | --- | --- | --- | --- | --- | --- | --- | --- | --- | --- | --- | --- |
| ID | Location | ID VET | Breed | Sex | Age | Vaccinated | CDV positive | Ocular | Respiratory | Digestive | Neurologic | Severe |
| CDV001 | RM | Vet 1 | English Setter | M | YA | 1 | 1 | 1 | 0 | 1 | 1 | 0 |
| CDV002 | RM | Vet 2 | Golden Retriever | M | YA | 1 | 1 | 1 | 1 | 0 | 1 | 0 |
| CDV003 | RM | Vet 3 | German Shepherd | M | MA | 1 | 0 | 0 | 1 | 1 | 0 | 1 |
| CDV004 | RM | Vet 4 | Mixed | F | MA | 1 | 0 | 1 | 1 | 0 | 0 | 0 |
| CDV005 | RM | Vet 1 | Mixed | F | Puppy | 0 | 1 | 0 | 1 | 1 | 1 | 0 |
| CDV006 | VA | Vet 5 | Mixed | M | Puppy | 1 | 1 | 1 | 1 | 1 | 1 | 0 |
| CDV007 | VA | Vet 5 | Mixed | F | Puppy | 0 | 0 | 1 | 0 | 1 | 0 | 0 |
| CDV008 | RM | Vet 1 | Mixed | M | YA | 0 | 1 | 1 | 1 | 1 | 0 | 1 |
| CDV009 | RM | Vet 6 | Mixed | M | Puppy | 1 | 0 | 0 | 0 | 1 | 0 | 0 |
| CDV010 | VA | Vet 5 | German Shepherd | M | Puppy | 1 | 0 | 0 | 0 | 1 | 0 | 0 |
| CDV011 | VA | Vet 5 | Mixed | F | MA | 0 | 0 | 1 | 1 | 1 | 1 | 1 |
| CDV012 | RM | Vet 1 | Mixed | F | Puppy | 0 | 1 | 1 | 1 | 1 | 1 | 0 |
| CDV013 | RM | Vet 7 | Mixed | F | Puppy | 0 | 0 | 0 | 0 | 1 | 0 | 1 |
| CDV014 | RM | Vet 7 | Mixed | M | Puppy | 0 | 0 | 0 | 0 | 1 | 0 | 1 |
| CDV015 | RM | Vet 1 | Mixed | F | Puppy | 0 | 1 | 1 | 1 | 0 | 0 | 0 |
| CDV016 | RM | Vet 3 | Mixed | M | MA | 1 | 1 | 1 | 1 | 1 | 1 | 0 |
| CDV017 | BI | Vet 8 | German Shepherd | M | Puppy |  | 0 |  |  |  |  |  |
| CDV018 | RM | Vet 3 | Mixed | F | MA | 1 | 1 | 1 | 0 | 1 | 0 | 0 |
| CDV019 | RM | Vet 7 | Mixed | F | YA | 0 | 1 | 1 | 1 | 1 | 1 | 0 |
| CDV020 | RM | Vet 7 | Mixed | M | MA | 0 | 1 | 0 | 0 | 1 | 1 | 0 |
| CDV021 | RM | Vet 7 | Poodle | M | MA | 1 | 0 |  |  |  |  |  |
| CDV022 | VA | Vet 9 | Yorkshire Terrier | M | MA | 1 | 0 | 1 | 0 | 0 | 1 | 0 |
| CDV023 | VA | Vet 9 | Mixed | M | YA | 0 | 1 | 1 | 1 | 0 | 1 | 1 |
| CDV024 | RM | Vet 10 |  | F |  |  | 0 |  |  |  |  |  |
| CDV025 | RM | Vet 16 |  | F |  |  | 0 |  |  |  |  |  |
| CDV026 | RM | Vet 7 | Mixed | M | Puppy | 1 | 0 | 1 | 0 | 1 | 0 | 1 |
| CDV027 | VA | Vet 9 | Mixed | M | Puppy | 1 | 1 | 1 | 1 | 1 | 1 | 1 |
| CDV028 | RM | Vet 7 | Mixed | M | MA | 1 | 0 | 0 | 1 | 1 | 0 | 1 |
| CDV029 | RM | Vet 7 | German Shepherd | F | MA |  | 0 | 1 | 0 | 0 | 0 | 0 |
| CDV030 | VA | Vet 9 | Mixed | F | Puppy | 0 | 1 | 0 | 1 | 1 | 0 | 1 |
| CDV031 | RM | Vet 11 | Mixed | M | MA | 1 | 1 | 1 | 1 | 1 | 1 | 0 |
| CDV032 | VA | Vet 9 | Mixed | F | Puppy |  | 0 | 0 | 1 | 1 | 0 | 0 |
| CDV033 | BI | Vet 12 |  | F |  |  | 0 |  |  |  |  |  |
| CDV034 | BI | Vet 12 |  | F |  |  | 1 |  |  |  |  |  |
| CDV035 | RM | Vet 7 | Teckel | F | MA | 1 | 0 | 0 | 1 | 0 | 0 | 0 |
| CDV036 | VA | Vet 9 | Mixed | F | YA | 0 | 0 | 0 | 0 | 0 | 1 | 0 |
| CDV037 | BI | Vet 12 |  | F |  |  | 0 |  |  |  |  |  |
| CDV038 | RM | Vet 1 | Poodle | M | MA | 1 | 1 | 1 | 0 | 1 | 1 | 0 |
| CDV039 | RM | Vet 13 | Mixed | F | MA | 1 | 0 | 0 | 0 | 0 | 1 | 0 |
| CDV040 | RM | Vet 1 | Chihuahua | F | MA | 1 | 1 | 1 | 1 | 1 | 1 | 1 |
| CDV041 | RM | Vet 1 | Poodle | M | Puppy | 0 | 1 | 1 | 1 | 1 | 0 | 0 |
| CDV042 | VA | Vet 9 | Mestizo | M | Puppy | 1 | 0 | 1 | 0 | 1 | 0 | 0 |
| CDV043 | VA | Vet 9 | Mestizo | F | Puppy | 1 | 1 | 1 | 0 | 1 | 0 | 0 |
| CDV044 | RM | Vet 1 | Pug | F | YA | 0 | 1 | 1 | 1 | 1 | 0 | 0 |
| CDV045 | RM | Vet 14 | Mestizo | F | Puppy | 1 | 1 | 1 | 1 | 1 | 1 | 1 |
| CDV046 | RM | Vet 13 | Mixed | F | MA | 1 | 0 | 0 | 0 | 1 | 1 | 0 |
| CDV047 | RM | Vet 13 | Mixed | M | YA | 0 | 1 | 1 | 1 | 1 | 1 | 0 |
| CDV048 | RM | Vet 15 | Mixed | F | Puppy | 0 | 1 | 1 | 1 | 0 | 1 | 0 |
| CDV049 | RM | Vet 7 | Mixed | M | YA | 0 | 1 | 0 | 1 | 1 | 1 | 0 |
| CDV050 | RM | Vet 7 | Mixed | F | Puppy | - | 1 | 1 | 0 | 1 | 0 | 1 |
| CDV051 | RM | Vet 7 | Mixed | F | Puppy | 0 | 1 | 1 | 0 | 1 | 0 | 1 |
| CDV052 | RM | Vet 7 | Mixed | F | Puppy | 0 | 1 | 0 | 0 | 1 | 0 | 1 |

RM: Metropolitana; VA: Valparaiso; BI: Bio Bio; M: male; F: female; YA: young adult (6 months-2 years); MA: mature adult (>2y); < 1: yes; 0: no.

**Table S2**. Vaccination status and RT-qPCR results of the suspected CD cases included in this study, Chile, 2022-2023.

|  |  | Vaccination status | | |  | RT-qPCR Ct values | | | | | |
| --- | --- | --- | --- | --- | --- | --- | --- | --- | --- | --- | --- |
| ID | Age stage | Vaccinated | Time | Outdated | CDV | N | O | ON | F | S | ORN |
| CDV001 | YA | Yes | > 1y | No | Positive | - | 21.2 | - | 25.9 | 27.4 | - |
| CDV002 | YA | Yes | 7m | Yes | Positive | - | - | 22.2 | - | - | - |
| CDV003 | MA | Yes | 4y11m | Yes | Negative | - | - | >40 | >40 | - | - |
| CDV004 | MA | Yes | 12y6m | Yes | Negative | - | - | >40 | >40 | - | - |
| CDV005 | Puppy | No |  |  | Positive | - | - | 23.01 | 16.22 | 17.17 | - |
| CDV006 | Puppy | Yes | 7m | - | Positive | - | - | >40 | >40 | 32.76 | - |
| CDV007 | Puppy | No |  |  | Negative | - | - | >40 | >40 | >40 | - |
| CDV008 | YA | No |  |  | Positive | - | - | 25.3 | 22.3 | 28.4 | - |
| CDV009 | Puppy | Yes | 4d | - | Negative | - | - | - | >40 | >40 | - |
| CDV010 | Puppy | Yes | 0d | - | Negative | - | - | - | >40 | >40 | - |
| CDV011 | MA | No |  |  | Negative | - | - | >40 | >40 | >40 | - |
| CDV012 | Puppy | No |  |  | Positive | - | - | 26.8 | - | - | - |
| CDV013 | Puppy | No |  |  | Negative | - | - | - | >40 | >40 | - |
| CDV014 | Puppy | No |  |  | Negative | - | - | - | >40 | >40 | - |
| CDV015 | Puppy | No |  |  | Positive | 22 | 23 | - | - | - | - |
| CDV016 | MA | Yes | 2y6m | No | Positive | - | - | 21.3 | 21 | 18 | - |
| CDV017 | Puppy | - |  |  | Negative | - | - | - |  | >40 | - |
| CDV018 | MA | Yes | 8m | No | Positive | - | - | 30.1 | 28.4 | 30.2 | - |
| CDV019 | YA | No |  |  | Positive | - | - | 26.27 | - | 30.28 | - |
| CDV020 | MA | No |  |  | Positive | - | - | - | 35.9 | - | - |
| CDV021 | MA | Yes | 4y4m | Yes | Negative | - | >40 | - | - | >40 | - |
| CDV022 | MA | Yes | 11m | No | Negative | - | >40 | - | >40 | >40 | - |
| CDV023 | YA | No |  |  | Positive | - | - | 36.9 | >40 | >40 | - |
| CDV024 |  | - |  |  | Negative | - | - | - | - | >40 | - |
| CDV025 |  | - |  |  | Negative | >40 | - | - | >40 | >40 | - |
| CDV026 | Puppy | Yes | 5d | - | Negative | - | - | >40 | >40 | >40 | - |
| CDV027 | Puppy | Yes | 5d | - | Positive | - | - | 18.8 | 20.5 | 19.3 | - |
| CDV028 | MA | Yes | 1y | No | Negative | - | - | - | >40 | >40 | - |
| CDV029 | MA | - |  |  | Negative | - | - | - | >40 | >40 | - |
| CDV030 | Puppy | No |  |  | Positive | - | - | >40 | >40 | 36.9 | - |
| CDV031 | MA | Yes | 2y2m | No | Positive | - | - | - | - | 31.9 | - |
| CDV032 | Puppy | - |  |  | Negative | - | - | >40 | >40 | >40 | - |
| CDV033 |  | - |  |  | Negative | - | - | - | - | >40 | - |
| CDV034 |  | - |  |  | Positive | - | - | - | - | 31 | - |
| CDV035 | MA | Yes | 2m | No | Negative | - | - | - | >40 | >40 | - |
| CDV036 | YA | No |  |  | Negative | - | - | >40 | >40 | >40 | - |
| CDV037 |  | - |  |  | Negative | - | - | >40 | >40 | >40 | - |
| CDV038 | MA | Yes | 2y2m | No | Positive | 30.2 | 28.6 | - | 17.5 | - | - |
| CDV039 | MA | Yes | 9y2m | Yes | Negative | - | - | >40 | - | - | - |
| CDV040 | MA | Yes | 6y3m | Yes | Positive | - | 25.2 | - | >40 | - | - |
| CDV041 | Puppy | No |  |  | Positive | - | 28.4 | - | 25 | - | - |
| CDV042 | Puppy | Yes | 3d | - | Negative | - | - | >40 | >40 | >40 | - |
| CDV043 | Puppy | Yes | 3d | - | Positive | - | - | >40 | >40 | 31.1 | - |
| CDV044 | YA | No |  |  | Positive | 25.5 | - | - | 20.2 | - | - |
| CDV045 | Puppy | Yes | 15d | - | Positive | - | - | 37 | 29.9 | - | - |
| CDV046 | MA | Yes | 1y4m | No | Negative | - | >40 | - | >40 | >40 | - |
| CDV047 | YA | No |  |  | Positive | - | >40 | - | >40 | 33.1 | - |
| CDV048 | Puppy | No |  |  | Positive | - | - | - | - | - | 24.8 |
| CDV049 | YA | No |  |  | Positive | - | - | 35.6 | 36.9 | - | 39.2 |
| CDV050 | Puppy | - |  |  | Positive | - | - | - | 39.1 | 35.5 | - |
| CDV051 | Puppy | No |  |  | Positive | - | 23.2 | - | 19.2 | 29 | - |
| CDV052 | Puppy | No |  |  | Positive | - | - | - | 33.2 | >40 | - |

YA: young adult (6 months-2 years); MA: mature adult (>2y); Vaccinated: at least one vaccine dose; Time: time since last vaccination in years (y), months (m) and days (d); Outdated: vaccination is overdue according to WSAVA 2024 vaccination guidelines; O: conjunctival swab; N: nasal swab; ON: pooled nasal and conjunctival swabs; F: fecal swab; S: blood with EDTA; ORN: urine.

**Table S3.** Amino acid substitutions on Chilean CDV field strains 2022-2023 compared to Onderstepoort reference strain, number of strains with the mutation, function/effect of the specific site, and parsimony informative sites.

| Position | Mutation | Number of strains | Change in polarity | Function/effect | Reference | Parsimony informative site |
| --- | --- | --- | --- | --- | --- | --- |
| 3 | P→S | 8 | Yes |  |  |  |
| 21 | T→S | 8 | No |  |  |  |
| 29 | G→E | 8 | Yes |  |  |  |
| 30 | H→Q | 8 | Yes |  |  |  |
| 33 | R→K | 3 | No |  |  | Yes |
| 50 | L→M | 8 | No |  |  |  |
| 51 | A→T | 3 | Yes |  |  | Yes |
| 68 | M→T | 2 | Yes |  |  |  |
| 78 | M→L | 1 | No |  |  |  |
| 78 | M→I | 2 | No |  |  | Yes |
| 83 | A→D | 2 | Yes |  |  | Yes |
| 103 | I→V | 3 | No |  |  | Yes |
| 141 | N→D | 1 | Yes |  |  |  |
| 145 | T→K | 8 | Yes |  |  |  |
| 146 | V→I | 7 | No |  |  | Yes |
| 155 | E→D | 8 | No |  |  |  |
| 156 | S→T | 8 | No |  |  |  |
| 162 | A→S | 8 | Yes |  |  |  |
| 172 | L→S | 2 | Yes |  |  | Yes |
| 186 | H→Y | 8 | Yes |  |  |  |
| 189 | S→N | 5 | No |  |  | Yes |
| 193 | T→I | 5 | Yes |  |  | Yes |
| 195 | V→M | 2 | No |  |  | Yes |
| 197 | K→R | 4 | No |  |  | Yes |
| 198 | V→I | 4 | No |  |  | Yes |
| 212 | R→K | 2 | No |  |  | Yes |
| 214 | S→T | 1 | No |  |  |  |
| 216 | V→I | 8 | No |  |  |  |
| 217 | I→T | 3 | Yes |  |  | Yes |
| 218 | N→T | 2 | No |  |  | Yes |
| 225 | D→G | 2 | Yes |  |  | Yes |
| 235 | V→M | 4 | No |  |  | Yes |
| 235 | V→I | 1 | No |  |  | Yes |
| 238 | D→Y | 6 | Yes | D238Y antigenic change in neutralizing epitope | (Bi et al., 2023; Wang et al., 2023) | Yes |
| 239 | I→T | 2 | Yes |  |  | Yes |
| 241 | R→G | 7 | Yes | R241G antigenic change in neutralizing epitope | (Bi et al., 2023; Wang et al., 2023) | |
| 241 | R→E | 1 | Yes |  |  |  |
| 246 | R→Q | 8 | Yes |  |  |  |
| 247 | E→Q | 8 | Yes |  |  |  |
| 262 | D→N | 4 | Yes |  |  | Yes |
| 263 | M→T | 1 | Yes |  |  |  |
| 276 | K→E | 8 | Yes |  |  |  |
| 278 | S→Y | 2 | No |  |  | Yes |
| 282 | V→I | 1 | No |  |  |  |
| 291 | T→A | 1 | Yes |  |  |  |
| 298 | E→D | 8 | No |  |  |  |
| 301 | T→S | 1 | No |  |  |  |
| 301 | T→N | 1 | No |  |  |  |
| 303 | L→S | 2 | Yes |  |  | Yes |
| 309 | S→N | 6 | No |  |  | Yes |
| 315 | I→V | 5 | No |  |  | Yes |
| 320 | L→M | 2 | No |  |  | Yes |
| 324 | W→G | 8 | No |  |  |  |
| 327 | P→S | 5 | Yes |  |  | Yes |
| 330 | H→Q | 8 | Yes |  |  |  |
| 331 | I→V | 8 | No |  |  |  |
| 341 | S→L | 5 | Yes |  |  | Yes |
| 342 | M→V | 8 | No |  |  |  |
| 343 | K→E | 8 | Yes |  |  |  |
| 349 | N→S | 1 | No |  |  |  |
| 353 | F→I | 5 | No |  |  | Yes |
| 353 | F→L | 1 | No |  |  | Yes |
| 367 | A→V | 8 | No | Putative antigenic residues | (Menezes et al., 2023) |  |
| 376 | G→N | 8 | Yes | Putative antigenic residues | (Menezes et al., 2023) |  |
| 386 | T→S | 5 | No | Putative antigenic residues | (Menezes et al., 2023) | Yes |
| 393 | A→T | 8 | Yes |  |  |  |
| 401 | R→G | 8 | Yes |  |  |  |
| 402 | Q→P | 1 | Yes |  |  |  |
| 415 | A→P | 8 | Yes |  |  |  |
| 417 | V→I | 8 | No |  |  |  |
| 424 | S→T | 1 | No |  |  |  |
| 430 | V→I | 5 | No |  |  | Yes |
| 443 | P→S | 3 | Yes |  |  | Yes |
| 445 | L→S | 3 | Yes |  |  | Yes |
| 446 | N→D | 8 | Yes |  |  |  |
| 456 | D→N | 8 | Yes |  |  |  |
| 459 | I→V | 8 | No |  |  |  |
| 460 | S→L | 8 | Yes | Nectin-4 binding residues | (Langedijk et al., 2011) |  |
| 467 | G→S | 8 | Yes |  |  |  |
| 470 | D→A | 1 | Yes |  |  |  |
| 470 | D→G | 1 | Yes |  |  |  |
| 475 | L→I | 8 | No |  |  |  |
| 488 | G→R | 2 | Yes |  |  | Yes |
| 500 | M→R | 8 | Yes | M500L decreased fusion efficiency | (Sattler et al., 2014) |  |
| 502 | R→K | 8 | No |  |  |  |
| 506 | I→A | 3 | Yes |  |  | Yes |
| 506 | I→T | 5 | No |  |  | Yes |
| 510 | I→L | 8 | No | Nectin-4 binding residues | (Langedijk et al., 2011) |  |
| 515 | T→I | 1 | Yes |  |  |  |
| 516 | Q→R | 2 | Yes |  |  | Yes |
| 517 | S→N | 8 | No |  |  |  |
| 518 | I→F | 8 | No |  |  |  |
| 522 | I→V | 5 | No | Nectin-4 binding residues | (Langedijk et al., 2011) | Yes |
| 530 | S→G | 1 | Yes | Lineage-related mutation; 530A lost fusion activity | (Nikolin et al., 2012; von Messling et al., 2005) | Yes |
| 530 | S→D | 2 | Yes | Lineage-related mutation; 530A lost fusion activity | (Nikolin et al., 2012; von Messling et al., 2005) | Yes |
| 530 | S→N | 3 | No | Lineage-related mutation; 530A lost fusion activity | (Nikolin et al., 2012; von Messling et al., 2005) | Yes |
| 542 | I→N | 2 | Yes |  |  | Yes |
| 549 | H→Y | 8 | Yes | Host-related mutation; 549A lost fusion activity | (McCarthy et al., 2007; von Messling et al., 2005) | |
| 572 | N→D | 8 | Yes |  |  |  |
| 586 | A→T | 8 | Yes |  |  |  |
| 598 | I→M | 5 | No |  |  | Yes |
| 599 | R→K | 3 | No |  |  | Yes |
| 599 | R→S | 2 | Yes |  |  | Yes |

**Table S4**. Vaccines against CDV available in Chile according to the Agricultural and Livestock Service (SAG), May 2024.

| N | Brand name | Manufacturer | Origin | Type | Strain |
| --- | --- | --- | --- | --- | --- |
| 1 | Nobivac DHPPi | Intervet international b.v. | Netherlands | Live | Onderstepoort |
| 2 | Canigen MHA2PPi/L | Virbac s.a. | France | Live | Lederle |
| 3 | Vanguard Plus 5/L | Zoetis inc. | USA | Live | N-CDV |
| 4 | Vanguard Plus 5CV/L | Zoetis inc. | USA | Live | N-CDV |
| 5 | Nobivac Puppy DP | Intervet international b.v. | Netherlands | Live | Onderstepoort |
| 6 | Nobivac DAPPvL2 | Intervet inc. Usa | USA | Live | Distemperoid (Onderstepoort) |
| 7 | Nobivac DAPPvL2+Cv | Intervet inc. Usa | USA | Live | Distemperoid (Onderstepoort) |
| 8 | Recombitek C6 CV | Boehringer ingelheim a h usa inc. | USA | Recombinant | CANARYPOX, CP258 |
| 9 | Recombitek C6 | Boehringer ingelheim a h usa inc. | USA | Recombinant | CANARYPOX, CP258 |
| 10 | RECOMBITEK C3 | Boehringer ingelheim a h usa inc. | USA | Recombinant | CANARYPOX, CP258 |
| 11 | Nobivac® DAPPv + L4 | Intervet inc. Usa | USA | Live | Distemperoid (Onderstepoort) |
| 12 | Vanguard Plus 5L4 | Zoetis inc. | USA | Live | N-CDV |
| 13 | Recombitek C8 | Boehringer ingelheim a h usa inc. | USA | Recombinant | CANARYPOX, CP258 |
| 14 | Nobivac Edge DAPPv + L4 | Intervet inc. Usa | USA | Live | Distemperoid (Onderstepoort) |
| 15 | Nobivac Puppy DP Plus | Intervet international b.v. | Netherlands | Live | Onderstepoort |
| 16 | VIOBIX C PUPPY ADVANCED | Bioveta a.s. | Czech Republic | Live | CDV BIO 11/A |
| 17 | VIOBIX C6R | Bioveta a.s. | Czech Republic | Live | CDV BIO 11/A |


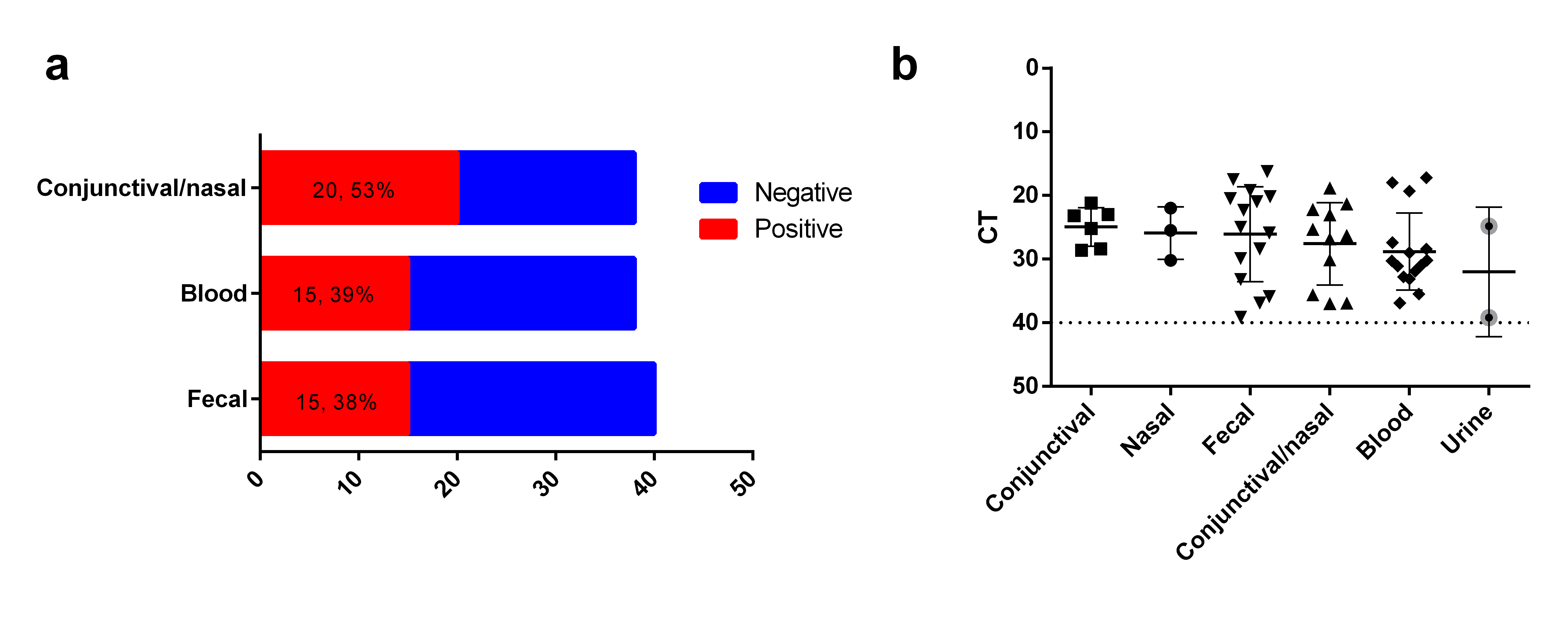


**Figure S1**.CDV RT-qPCR positivity according to sample type in suspected CD cases, Chile, 2022-2023. Overall CDV positivity of 118 samples from 52 suspected CD cases included in this study; conjunctival/nasal category included pooled and individual samples.

**Figure S2**. CDV positivity according to vaccination status in >6-month-old dogs, Chile, 2022-2023. According to Fisher's exact test (p-value = 1), CDV positivity is not associated with vaccination status in >6-month-old dogs.


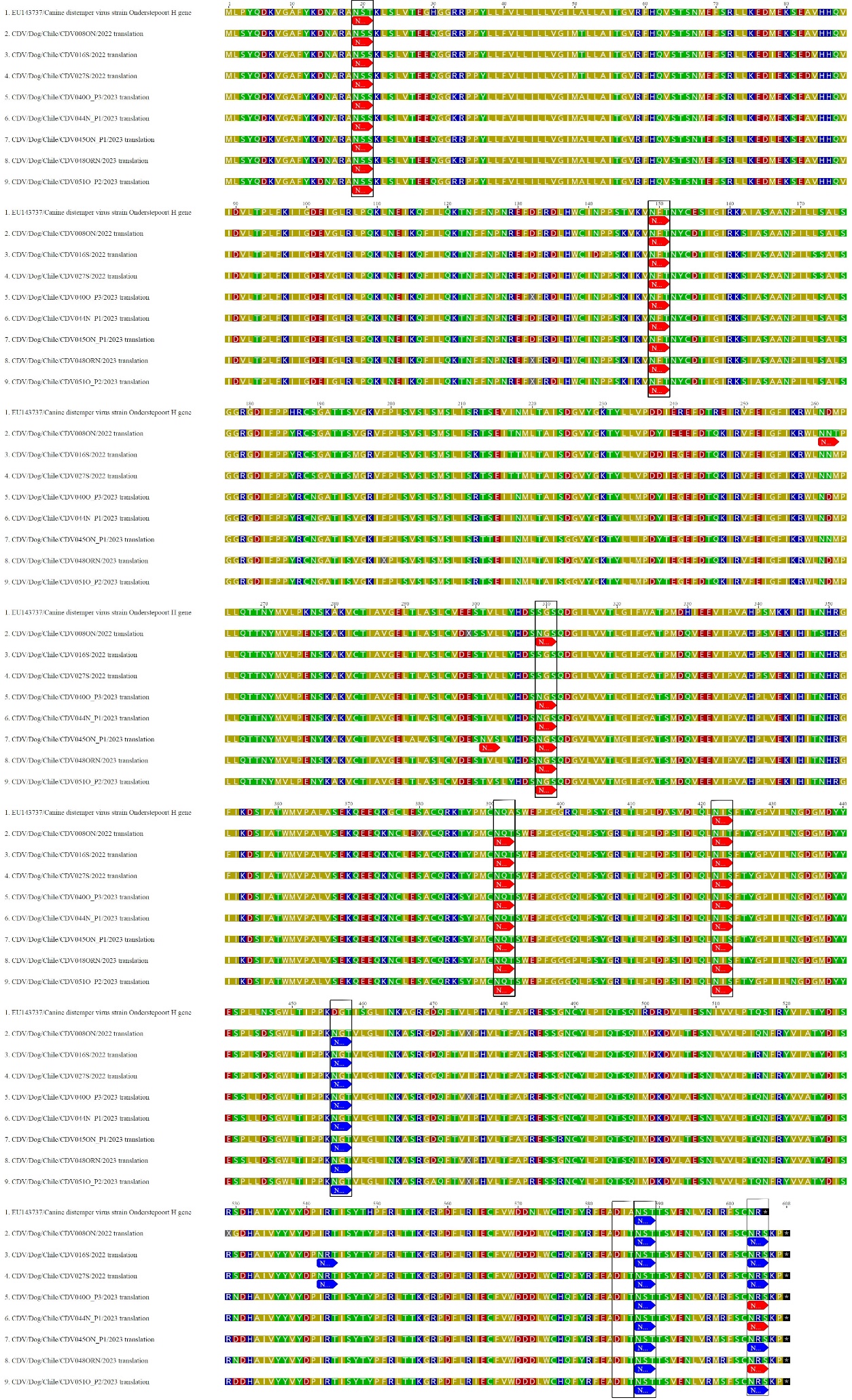


**Figure S3**. Putative N-linked glycosylation sites predicted in Chilean and Onderstepoort CDV strains amino acid sequences using NetNGlyc 1.0. Asn-Xaa-Ser/Thr motifs are highlighted in blue. Asparagines predicted to be N-glycosylated are highlighted in red. Glycosylation sites frequently found in different CDV lineages according to Bhatt et al., 2019, are labeled in black rectangles.
